# Ultrasound Assessment of the Anterolateral Ligament of the Knee: A Narrative Review of Current Evidence, Interpretative Limitations, and Clinical Context

**DOI:** 10.3390/diagnostics16071084

**Published:** 2026-04-03

**Authors:** Paweł Niewiadomy, Katarzyna Szuścik-Niewiadomy

**Affiliations:** 1 Department of Balneoclimatology and Biological Regeneration, School of Health Sciences in Katowice, Medical University of Silesia in Katowice, 40-752 Katowice, Poland; pniewiadomy@sum.edu.pl; 2 Department of Adapted Physical Activity and Sport, School of Health Sciences in Katowice, Medical University of Silesia in Katowice, Medyków 8, 40-752 Katowice, Poland

**Keywords:** anterolateral ligament, anterior cruciate ligament, diagnostic limitations, musculoskeletal ultrasound, rotational instability

## Abstract

Rotational knee instability remains a relevant clinical problem, particularly in patients with anterior cruciate ligament injury, and has renewed interest in the anterolateral ligament (ALL) as a contributing structure. This narrative review critically synthesizes current anatomical, biomechanical, and ultrasonographic evidence regarding the ALL, with emphasis on the interpretative capabilities and limitations of musculoskeletal ultrasound. Available data indicate that ultrasound allows anatomical identification of the ALL, primarily in asymptomatic populations, but does not support its use as a standalone diagnostic tool for ALL injury. Dynamic ultrasound approaches remain observational, non-standardized, and lack clinical validation. Ultrasound may be considered only as a complementary modality within a clearly defined clinical context.

## 1. Introduction

Rotational instability of the knee is a well-documented clinical problem, particularly in patients with anterior cruciate ligament (ACL) injury. Despite the widespread use of clinical tests, including the pivot shift, and the availability of imaging modalities, the mechanisms responsible for excessive tibial rotation relative to the femur remain incompletely understood and continue to be the subject of intensive investigation. Over the past decade, particular attention has been directed toward the anterolateral ligament (ALL), which has been re-described as a distinct anatomical structure in the context of rotational knee stability [1,2].

Since this renewed interest in the ALL, numerous anatomical and histological studies have confirmed the presence of this structure while simultaneously demonstrating substantial variability in its course, insertions, and relationships with adjacent anterolateral structures, including the joint capsule and layers of the iliotibial band region. This anatomical variability has important implications for imaging interpretation and contributes to the risk of misidentification of anterolateral structures [3,4].

Biomechanical investigations indicate that the ALL does not function as an isolated stabilizer but rather serves as a supportive structure contributing to the control of internal tibial rotation, operating in functional conjunction with the ACL and other lateral knee structures. In practical terms, this means that neither imaging findings nor functional tests can be attributed to a single anatomical structure without considering the systemic nature of rotational knee stability [5,6].

In parallel with anatomical and biomechanical research, attempts have been made to visualize the anterolateral ligament of the knee using musculoskeletal ultrasound. Cadaveric studies have demonstrated high sensitivity of ultrasound for identifying the ALL as an anatomical structure, while emphasizing that the assessment of ligament injury and its diagnostic value require separate clinical validation [7]. Observations in healthy volunteers have further confirmed that ultrasonographic visualization of the ALL is feasible; however, the clarity of identification and image quality may vary considerably between individuals [8].

Subsequent studies have focused on the standardization of examination protocols and the assessment of measurement reliability. Available data suggest that agreement in ALL identification and the repeatability of selected measurements are moderate to high in asymptomatic populations, with reliability being strongly dependent on the parameter assessed—higher for linear dimensions and distances, and markedly lower for thickness measurements. Importantly, technical reliability alone does not determine the diagnostic value of ultrasound in the assessment of anterolateral knee pathology [9,10].

The literature has also described approaches referred to as dynamic ultrasonographic assessment of the ALL, involving observation of anterolateral structures during provoked knee rotation and the use of repeatable anatomical landmarks, particularly at the tibial insertion. However, these approaches have not been supplemented with validated diagnostic criteria enabling clinical assessment of ALL injury [11]. From a clinical perspective, consensus statements emphasize that rotational knee instability and related therapeutic decisions should be considered in an integrated manner, incorporating clinical symptoms, functional test findings (including the pivot shift), the context of ACL injury, and associated osseous lesions such as the Segond fracture. Within this framework, the ALL is regarded as a component of the broader anterolateral complex rather than an isolated counterpart of primary stabilizing ligaments [12].

The aim of the present study is to provide a structured and critical synthesis of the current state of knowledge regarding the ALL from the perspective of musculoskeletal ultrasound by addressing: (1) what is known and the sources of that knowledge (anatomical and biomechanical evidence); (2) what ultrasound can realistically achieve at the present stage (identification, protocols, and reliability); (3) the key interpretative limitations (anatomical variability, operator dependence, lack of diagnostic criteria and clinical validation); and (4) the evidence gaps that must be addressed before definitive clinical conclusions can be drawn.

Given the variability of the available evidence, the lack of standardized diagnostic criteria, and the multidimensional nature of the problem, a narrative review approach was adopted to provide a clinically oriented synthesis of current knowledge.

## 2. Methodological Approach

This manuscript is a narrative review aimed at providing a clinically oriented synthesis of current knowledge regarding the ultrasound assessment of the anterolateral ligament (ALL) of the knee.

The literature was selected based on its relevance to the anatomical, biomechanical, and imaging aspects of the ALL, with particular emphasis on ultrasonographic assessment and its clinical interpretation.

Sources were identified through searches in PubMed and related databases using combinations of keywords such as “anterolateral ligament”, “knee”, “ultrasound”, and “imaging”.

## 3. The Anterolateral Ligament of the Knee: Anatomical, Biomechanical, and Imaging Background in Light of Current Evidence

### 3.1. Definition and Anatomical Variability of the Anterolateral Ligament

The anterolateral ligament of the knee (ALL) has been re-described in the anatomical literature as a structure located within the anterolateral compartment of the knee joint, extending between the region of the lateral femoral epicondyle and the anterolateral surface of the tibia, in close proximity to Gerdy’s tubercle. Cadaveric studies have demonstrated, however, substantial variability with respect to the presence, course, width, and anatomical relationships of the ALL with other lateral knee structures, which has from the outset generated debate regarding its precise anatomical definition [1,2,5].

Anatomical investigations have emphasized that the ALL does not consistently present as a clearly delineated, independent ligamentous structure. In a proportion of specimens, its fibers exhibit close continuity with the joint capsule, the iliotibial band, or structures collectively referred to as the anterolateral complex, thereby complicating the clear demarcation of ligament boundaries, particularly at the femoral attachment site [2,5]. This anatomical variability has important implications for the interpretation of imaging studies, in which in vivo identification of the ALL remains inherently dependent on the anatomical definition adopted. The presence of the anterolateral ligament has also been confirmed during fetal development, with anatomical and histological characteristics comparable to those observed in adults [13].

### 3.2. Biomechanical and Clinical Significance of the Anterolateral Ligament of the Knee

Anatomical and biomechanical studies consistently indicate that the anterolateral ligament of the knee does not function as an isolated stabilizer of the knee joint but rather represents a component of a complex system responsible for the control of rotational stability, acting in close functional conjunction with the anterior cruciate ligament and other structures of the lateral compartment of the knee [1,3,4]. Variability in the course of the ALL, its attachment sites, and its relationships with the joint capsule and the iliotibial band has been repeatedly confirmed in cadaveric and histological studies, with direct implications for the interpretation of both biomechanical and imaging investigations [1,4].

In vitro biomechanical studies have demonstrated that the contribution of the ALL to the control of internal tibial rotation is secondary in nature and is closely dependent on the knee flexion angle as well as the functional status of the ACL. Parsons et al. showed that sectioning of the ALL results in a moderate increase in internal tibial rotation; however, this effect becomes substantially more pronounced only in the presence of concomitant ACL deficiency, supporting the role of the ALL as a secondary stabilizing structure [5]. Similar conclusions were reported by Rasmussen et al., who, using a robotic testing system, demonstrated that the ALL does not act as a primary rotational stabilizer but rather provides supportive restraint in the setting of central ligament injury [6].

A clinically relevant aspect of rotational knee instability is the association between anterolateral structures and avulsion injuries, particularly the Segond fracture. Dodds et al. demonstrated that the location and mechanism of this type of fracture are closely related to the course and tension of anterolateral structures, including the ALL, indicating their involvement in injuries characterized by a pronounced rotational component [2]. These observations support the concept of the anterolateral complex as a functional unit, in which the biomechanical consequences of injury cannot be attributed to a single anatomical structure.

From a clinical perspective, the key manifestation of rotational instability is a positive pivot shift, which is widely regarded as the result of complex biomechanical disturbances involving the ACL as well as lateral and anterolateral knee structures. Biomechanical studies and clinical analyses indicate that a high-grade pivot shift does not reflect isolated injury to the ALL but rather arises from the cumulative disruption of the entire stabilizing system of the knee [5,6]. Consensus statements emphasize that interpretation of the pivot shift and related therapeutic decisions should account for the overall mechanism of rotational instability rather than relying on assessment of a single anatomical structure [12].

This biomechanical and clinical perspective has direct implications for the interpretation of imaging findings, including musculoskeletal ultrasound. The systemic nature of rotational knee stability and the secondary role of the ALL justify caution in attempts at its isolated assessment and provide the rationale for the critical analysis of the capabilities and limitations of ultrasonographic evaluation of the ALL, as discussed in the subsequent sections of this manuscript.

### 3.3. Ultrasonographic Anatomy of the Anterolateral Ligament of the Knee in In Vivo Studies

The first reports addressing in vivo ultrasonographic identification of the anterolateral ligament of the knee focused on the feasibility of anatomical visualization of this structure using high-frequency linear transducers and appropriately selected anatomical landmarks within the anterolateral compartment of the knee. The objective of these studies was not the diagnosis of ligament injury, but rather the determination of whether the ALL could be recognized as a distinct anatomical structure on ultrasound imaging [7].

Cavaignac et al. described a technique for localizing the ALL based on identification of its tibial portion in the vicinity of Gerdy’s tubercle, followed by proximal tracking of a band with a fibrillar echotexture along the anterolateral aspect of the knee. The authors emphasized the spatial relationships between the ALL, the iliotibial band, and the joint capsule, which may hinder clear delineation of individual structural boundaries on ultrasound images. These investigations were conducted in asymptomatic individuals and were purely anatomical in nature [7].

Oshima et al. expanded upon these observations by analyzing the feasibility of identifying individual segments of the ALL. They demonstrated that the tibial portion of the ligament could be visualized consistently, whereas identification of the femoral portion was substantially limited and achievable only in a subset of examined subjects. These limitations were attributed to the small dimensions of the ligament, its anatomical variability, and its close proximity to the iliotibial band and joint capsule, which promotes overlap of echogenic structures on ultrasound imaging [8].

Available in vivo studies indicate that ultrasonographic identification of the ALL relies primarily on assessment of its echotexture, course, and spatial relationships with adjacent tissues, without the use of validated quantitative criteria or reference to comparative imaging modalities in clinical populations. Descriptions of examination protocols highlight the importance of appropriate lower limb positioning and transducer orientation; however, they are characterized by substantial technical variability, which limits direct comparison of findings across studies [7,8].

Some publications have also reported the possibility of observing changes in the position and tension of anterolateral structures during knee motion. These descriptions, however, are qualitative and exploratory in nature, lacking standardized procedures and clearly defined interpretative criteria [11,14,15]. A detailed analysis of so-called static and dynamic approaches, along with their interpretative limitations, is presented in Section 3.

## 4. Clinical Interpretation and Limitations of Ultrasonographic Assessment of the Anterolateral Ligament of the Knee: An Evidence-Based Synthesis

### 4.1. Interpretation of Ultrasonographic Assessment of the Anterolateral Ligament in Light of Quantitative Data (Static Assessment)

Analysis of available ultrasonographic studies indicates that the feasibility of in vivo identification of the anterolateral ligament of the knee (ALL) is high; however, this primarily applies to asymptomatic populations and refers to anatomical identification of the structure rather than to clinical diagnosis of ligament injury. In studies conducted in healthy volunteers, the rate of successful anatomical ALL identification exceeded 90%, confirming that the ligament can be visualized under favorable imaging conditions and with the use of predefined anatomical landmarks [9].

At the same time, quantitative data clearly demonstrate that the reliability of ultrasonographic assessment of the ALL is strongly dependent on the specific morphological parameter evaluated. The highest repeatability was reported for structural identification itself (ICC ≈ 0.90) and for measurements of ligament length (ICC ≈ 0.83), whereas reliability of thickness measurements was notably lower (ICC ≈ 0.75), suggesting limited reliability of certain ALL characteristics to objective ultrasonographic evaluation [9].

Analyses by Kandel et al. further confirmed that variability in reliability is influenced not only by the type of parameter measured but also by measurement location and operator experience. For selected ligament dimensions, such as length and width, good to very good intraclass correlation coefficients were reported (ICC ≈ 0.80–0.88), while other parameters—particularly thickness—demonstrated very low repeatability, with ICC values as low as approximately 0.35 [10]. These findings indicate substantial limitations in the comprehensive and uniform ultrasonographic assessment of the entire course of the ALL particularly beyond the distal (tibial) portion.

An additional factor limiting quantitative interpretation is the unequal feasibility of assessing individual ligament segments. Available data indicate that measurements of the distal (tibial) portion of the ALL demonstrate greater repeatability than observations of the proximal (femoral) segment, which affects overall examination reliability and complicates comparison of results across studies [8,10].

Only a limited number of studies have addressed ultrasonographic assessment of the ALL in patients with acute anterior cruciate ligament injury, focusing on static evaluation of ligament morphology and comparison with magnetic resonance imaging (MRI). Cavaignac et al. explored the feasibility of identifying ultrasonographic features suggestive of ALL injury in patients with ACL rupture and reported a partial correspondence between ultrasound findings and MRI observations, while emphasizing the absence of clearly defined diagnostic criteria allowing independent clinical interpretation [11]. Similarly, Bilfeld et al. evaluated the appearance of the ALL on ultrasound and MRI in patients with acute ACL injury, reporting relevant discrepancies between imaging modalities and highlighting interpretative limitations related to the absence of a reference standard rather than to a definitive diagnostic framework [16].

It should be emphasized that the study by Bilfeld et al. comparing ultrasonography with magnetic resonance imaging in the assessment of the ALL does not provide data enabling direct comparison of the reliability of the two imaging modalities. MRI served as a clinical reference imaging tool; however, no formal agreement analyses or quantitative comparisons of reliability coefficients between modalities were performed. Consequently, based on these data, it is not possible to determine conclusively which imaging modality demonstrates greater reliability for the assessment of ALL injury [16].

### 4.2. Dynamic Ultrasonographic Assessment of the Anterolateral Ligament: Proposed Methodological Concepts and Sources of Heterogeneity

In some publications, attempts have been made to assess the anterolateral ligament and other anterolateral knee structures under conditions of joint motion, referring to such approaches as dynamic ultrasonographic assessment. In the present manuscript, this term is used to describe observations of anterolateral structures during knee motion or provoked rotational maneuvers performed without validated examination protocols and without clearly defined interpretative criteria that would allow these procedures to be regarded as diagnostic tests.

#### 4.2.1. Concept 1: Passive Tibial Rotation with Femoral Stabilization—A Technical Approach

The most detailed description of dynamic ultrasonographic assessment of the ALL was provided by Cavaignac et al. in a technically oriented study. The authors proposed evaluation of anterolateral structures with the patient in the supine position, the knee flexed to approximately 90°, the femur stabilized, and internal rotation of the lower leg passively induced. The assessment focused on observation of changes in tissue tension within the distal portion of the ALL, particularly near its tibial attachment in the vicinity of Gerdy’s tubercle, using repeatable anatomical landmarks and transducer orientation aligned with the course of the fibrous structures. In some observations, changes in Doppler signal within the inferior lateral genicular artery were described as a potential surrogate marker of anterolateral tissue tension. However, although the authors proposed clinical implications of these observations, the study did not provide quantitative data on the reliability of dynamic assessment or its validated relationship to the severity of rotational instability or clinical test outcomes, limiting its contribution to the description of a technical concept rather than a diagnostic test [11].

#### 4.2.2. Concept 2: Provocation of Anterolateral Structure Tension at a Defined Flexion Angle—Biomechanical Rationale

Elements of functional provocation of ALL behavior have also been described in cadaveric ultrasonographic investigations. In the study by Capo et al., anterolateral structures were assessed using ultrasound with the knee flexed to approximately 30–60° and internal tibial rotation applied to accentuate structural tightening, with subsequent anatomical correlation on dissection. Although this investigation was not clinical in nature and did not include quantitative biomechanical measurements, it provided a rationale for the use of internal rotation as a maneuver to enhance visualization of anterolateral structures and demonstrated that the ultrasonographic appearance of the ALL is influenced by knee flexion angle. The authors did not, however, present data enabling assessment of diagnostic performance or repeatability of such observations in in vivo ultrasonographic examination [17].

#### 4.2.3. Sources of Heterogeneity and Interpretative Limitations

A major source of heterogeneity among proposed dynamic approaches lies in differences in technical study design. The described maneuvers vary substantially across studies with respect to initial knee position (approximately 30° versus 90° of flexion), methods of femoral and tibial stabilization, the nature of movement (passive versus active), and the range and method of rotational provocation [11,14,17]. Additional variability relates to the anatomical region under observation (distal versus proximal ligament portions), transducer orientation, and methods of documenting ultrasonographic findings, which precludes direct comparison of results between publications [11,15].

Importantly, available studies have not demonstrated that dynamic ultrasonographic assessment of the ALL provides greater informational value than static evaluation. No data have been presented regarding the reliability of dynamic observations, their correlation with the severity of rotational instability, clinical test results (e.g., the pivot shift), or other imaging modalities in a manner that would allow meaningful assessment of their diagnostic relevance [14,15].

## 5. Discussion

The synthesis of available anatomical, biomechanical, and ultrasonographic evidence indicates that the anterolateral ligament of the knee represents a structure of relevant, yet secondary importance in the control of rotational stability, functioning in close conjunction with the anterior cruciate ligament and other components of the anterolateral complex. This biomechanical role determines both the potential capabilities and the inherent limitations of its imaging assessment.

The present synthesis supports the view that the anterolateral ligament should be interpreted as a component of the anterolateral complex rather than an isolated stabilizer.

Current applications of musculoskeletal ultrasound allow for anatomical identification of the ALL and evaluation of selected morphological features; however, the scope of these observations is constrained by anatomical variability, operator dependence, and the absence of validated interpretative criteria, particularly in patient populations following ACL injury. Recent meta-analyses suggest that procedures addressing the anterolateral structures may improve rotational stability and reduce graft failure rates following ACL reconstruction. However, these findings are derived from surgical and clinical outcome studies and do not provide validated imaging-based criteria for the assessment of the anterolateral ligament [18,19]. Available data also do not permit a definitive comparison of the reliability of ultrasonography and magnetic resonance imaging in the diagnosis of ALL injuries.

With regard to dynamic assessment, the existing literature indicates that only conceptual and descriptive attempts have been made to observe the behavior of anterolateral structures during knee motion. The lack of standardized examination protocols, quantitative parameters, and clinical validation currently precludes consideration of these approaches as diagnostic tools. At the same time, the identified evidence gaps delineate directions for future research, particularly in the development of methodologically structured ultrasonographic protocols grounded in the biomechanics of the knee joint as an integrated system. These observations are consistent with recent bibliometric analyses, which highlight the rapid expansion of research on the anterolateral ligament, while simultaneously indicating the need for further development and standardization of imaging techniques and diagnostic frameworks [20].

**Figure 1:**
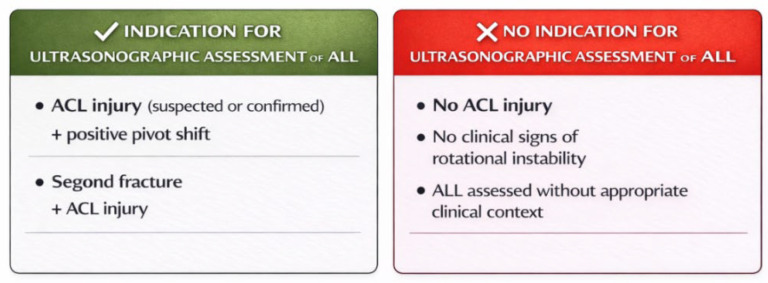
Clinical indications and non-indications for ultrasonographic assessment of the anterolateral ligament of the knee (ALL) based on current evidence.

**Figure 2:**
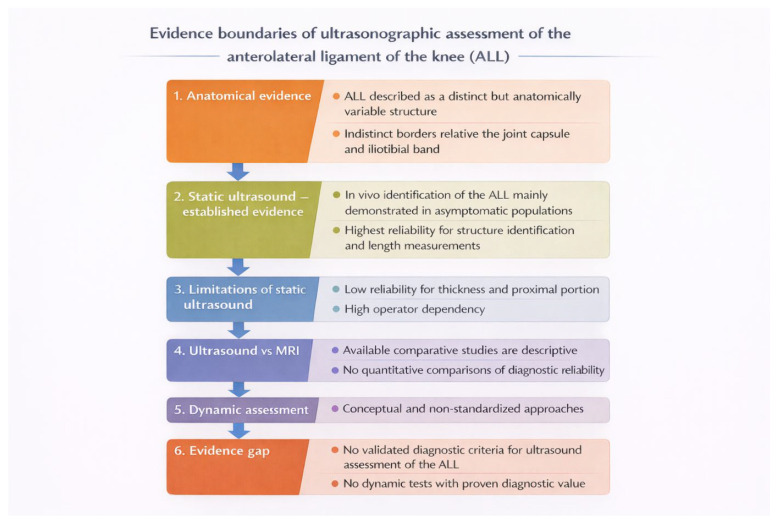
Evidence-based boundaries of ultrasonographic assessment of the anterolateral ligament of the knee (ALL).

These conclusions are synthetically illustrated in Figure 1 and Figure 2, which organize both the clinical context of musculoskeletal ultrasound applications and the current evidence-based boundaries of its use in the assessment of the anterolateral ligament.

A structured overview of key anatomical, biomechanical, and ultrasonographic studies informing the interpretation and limitations of ALL assessment is provided in Supplementary Table S1. Schematic overview of confirmed findings and methodological limitations related to static and dynamic ultrasonographic evaluation of the ALL.

## 6. Conclusions

1. The anterolateral ligament of the knee (ALL) functions as a secondary structure in the control of rotational stability and operates in close functional conjunction with the anterior cruciate ligament (ACL) and other components of the lateral compartment of the knee, which fundamentally limits the possibility of its isolated interpretation in imaging studies.
2. Musculoskeletal ultrasound allows for anatomical identification of the ALL and assessment of selected morphological features; however, available studies have been conducted predominantly in asymptomatic populations and do not provide validated criteria that would allow independent diagnosis of ALL injury.
3. The reliability of ultrasonographic assessment of the ALL is parameter-dependent, being highest for structural identification and selected linear measurements, and substantially lower for other features (e.g., thickness), with a clear influence of examination protocol and operator experience on measurement repeatability.
4. Approaches described in the literature as dynamic ultrasonographic assessment are currently observational and conceptual in nature, lack standardized procedures and clearly defined interpretative criteria, and cannot currently be regarded as diagnostic tests or interpreted analogously to established ligamentous tests.
5. In clinical practice, ultrasonographic assessment of the ALL may be considered only as a complementary modality, applied within a strictly defined clinical context, such as suspected significant rotational knee instability in the setting of ACL injury or anterolateral injuries associated with a Segond fracture.
6. Future research should focus on the development of methodologically structured ultrasonographic protocols, including attempts to standardize and validate dynamic assessments, with clearly defined examination conditions, interpretative criteria, and reference to whole-joint knee biomechanics and clinical data.

## Data Availability

No new data were created or analyzed in this study.

## Supplementary Materials

The following supporting information can be downloaded at: https://www.mdpi.com/article/10.3390/diagnostics16071084/s1, Table S1. Key studies on the anterolateral ligament (ALL) of the knee in the context of anatomy, biomechanics, and musculoskeletal ultrasonography—a critical synthesis.

## Abbreviations

The following abbreviations are used in this manuscript:

ALL	The anterolateral ligament
ACL	Anterior cruciate ligament
ICC	Intraclass correlation coefficient
MRI	Magnetic resonance imaging
ITB	Iliotibial band
