# Supplementary material for: Ultrasound Assessment of the Anterolateral Ligament of the Knee: A Narrative Review of Current Evidence, Interpretative Limitations, and Clinical Context
Source: Diagnostics (Basel). 2026 Apr 3;16(7):1084. doi: 10.3390/diagnostics16071084 (PMC13073188; doi:10.3390/diagnostics16071084)
Supplement: Supplementary file 1 [file diagnostics-16-01084-s001.zip › diagnostics-4190958-supplementary.pdf]

Supplementary Table S1. Key studies on the anterolateral ligament (ALL) of the knee in the context of anatomy, biomechanics, and musculoskeletal ultrasonography – a critical synthesis.

The table includes selected key studies that directly inform the anatomical, biomechanical, and ultrasonographic feasibility and interpretative limitations of ALL assessment, rather than an exhaustive list of all published studies.

Author (year)	Study type / population	Primary aim	Scope of ALL assessment	Dynamic assessment	What authors consider feasible with ultrasound	Main limitations reported by authors
Claes et al. (2013)	Cadaveric anatomical study	Re-description of ALL anatomy	Course, insertions, relationship with ITB and joint capsule	No	—	Anatomical variability; indistinct structural borders
Dodds et al. (2014)	Cadaveric + biomechanical	ALL length changes and association with Segond fracture	Tibial insertion; length changes during knee motion	Indirect (robotic)	—	ALL is not a primary stabilizer
Helito et al. (2013)	Anatomy + histology	Structural characterization of the ALL	Relationship with ITB and joint capsule	No	—	Difficult separation from adjacent structures
Caterine et al. (2015)	Cadaveric anatomical study	Anatomical variability of the ALL	Location and number of insertions	No	—	Lack of anatomical uniformity
Parsons et al. (2015)	In vitro biomechanics	Biomechanical role of the ALL	Interaction with ACL in rotational stability	Yes (robotic)	—	No isolated stabilizing function
Rasmussen et al. (2016)	Robotic biomechanical study	Control of tibial rotation	Role of ALL and ITB	Yes	—	ALL acts as a secondary stabilizing structure
Cavaignac et al. (2016)	Ultrasound + cadaveric correlation	Identification of the ALL using ultrasound	Anatomical landmarks; tibial insertion	No	Anatomical identification of the ALL	No validation of diagnostic utility
Oshima et al. (2016)	Ultrasound – healthy volunteers	Visualization of the ALL on ultrasound	Superficial course relative to joint capsule	No	Visualization of the ALL in selected subjects	Image variability; no clinical data

**Author (year)**	**Study type / population**	**Primary aim**	**Scope of ALL assessment**	**Dynamic assessment**	**What authors consider feasible with ultrasound**	**Main limitations reported by authors**
Argento et al. (2018)	Ultrasound – healthy subjects	Reliability of ALL identification	Standardized ultrasound protocol	No	Moderate to high inter- and intraobserver reliability	Healthy population only
Cavaignac et al. (2017)	Technical note (ultrasound)	Description of an ultrasound protocol for ALL	ITB → Gerdy's tubercle → posterior region	Yes (observational)	Qualitative assessment of tissue tension	No diagnostic criteria
Kandel et al. (2019)	Ultrasound + cadaveric	Reliability of an ultrasound protocol	Landmarks; measurement reproducibility	Limited	Technical standardization possible	Strong operator dependence
Sonnery-Cottet et al. (2019)	Expert consensus	Clinical relevance of the ALL	ALL in rotational knee instability	—	—	No definitive diagnostic test
Santoso et al. (2020)	Narrative review	Update on current ALL research	Anatomy, biomechanics, clinical relevance	—	—	No new diagnostic criteria
Littlefield et al. (2021)	Systematic review	Synthesis of ALL evidence	Anatomy, biomechanics, clinical outcomes	—	—	Lack of diagnostic consensus; no validated US criteria
